# Herbal Medicine Development: Methodologies, Challenges, and Issues

**DOI:** 10.1155/2019/4935786

**Published:** 2019-10-09

**Authors:** Juntra Karbwang, Francis P. Crawley, Kesara Na-Bangchang, Cecilia Maramba-Lazarte

**Affiliations:** ^1^Nagasaki University, Nagasaki, Japan; ^2^Good Clinical Practice Alliance-Europe (GCPA) and Strategic Initiative for Developing Capacity in Ethical Review-Europe (SIDCER), Brussels, Belgium; ^3^Thammasat University, Bangkok, Thailand; ^4^Institute of Herbal Medicine, National Institutes of Health, University of the Philippines, Manila, Philippines

Herbal medicines have been used in traditional medical practices for centuries. While practitioners of herbal medicines are often comfortable with the application of such medicines and are also largely convinced by the results they experience with patients, the scientific validity of herbal medicines in research and development is still often questioned. This is partly due to the lack of quality control, identification, and standardization of chemical compounds and drug formulations, as well as the challenges of applying the same methodologies across dissimilar medicinal products. The development of herbal medicine within the framework of evidence-based medicine is relatively new. Applying the methodologies of Western-based pharmaceutical sciences to herbal medicines presents the research community with a complex set of challenges. However, it is essential that herbal medicine research design and conduct be scientifically and ethically sound, while also taking into account the medical philosophies and practices that accompany the use of traditional herbal medicine.

Our special issue is focused on the application of modern technologies and methodologies in herbal medicine research and development using the accepted Western scientific and ethical standards. In the last decades, there has been significant progress in advancing traditional herbal medicine research and development, particularly in Asia, where there is an abundance of medicinal plants. As this issue will illustrate, the application of modern technologies and methodologies can have a significant impact on the scientific validity, quality improvement, and standardization of herbal medicines.

In the study of L. Yu* et al.,* the extraction of the bioactive ingredient glycyrrhizic acid from* Glycyrrhiza glabra* was successfully optimized using a mathematical modeling approach. The plant is one of the most widely used traditional medicines in China, mainly for invigorating the spleen, replenishing qi, and clearing away heat and toxic substances to treat diseases such as the weakness of the spleen and stomach, coughs, and phlegm. The authors demonstrated that the combinational method of genetic algorithm and artificial neural networks provides a reliable and accurate strategy for designing and optimizing glycyrrhizic acid extraction from* Glycyrrhiza glabra*.

Q. He* et al.* investigated the association between the proangiogenic mechanisms of Long-Zhi Decoction (LZD) medicated serum on cellular autophagy. The promotional effect of LZD on angiogenesis was examined* in vitro* using human umbilical vein endothelial cells (HUVECs). Proliferation, migration, and tube formation were assessed in a cell-based assay. A transmission electron microscope was used in the measurement of autophagosomes to determine the extent of autophagy. Immunofluorescence and Western blot were used to determine the autophagy-related proteins of LC3-ll and Beclin-1. The results showed that the LZD-medicated serum increased autophagosomes and the autophagic protein expressions of LC3-ll and Beclin-1. The LZD-medicated serum also promoted the proliferation, tube formation, and invasion of HUVECs. This may suggest that LZD promotes angiogenesis by increasing cellular autophagy.

P. Liang* et al.* conducted a preliminary study in a transgenic strain of mice with adenocarcinoma of the mouse prostate (TRAMP) to evaluate the efficacy of the Deep Immune® (DI) as a prevention strategy to control the progression of prostate cancer. DI is a commercial natural health product consisting of a mixture of eight medicinal herbs. It was shown to stimulate phagocytosis and expression of a panel of inflammatory mediators (C4b, CXCL3, lymphotoxin, NOS2, TLR1, TNF, and TNFSF14) in cultured macrophages* in vitro.* In addition, it also increases the tumor-killing ability of both macrophages and TRAMP mouse splenocytes. Daily intake of this herbal product significantly prevented prostate cancer progression, suggesting the possible effectiveness of the immunostimulant herbal products in the prevention of the progression of prostate cancer.

G. Kasparaviciene et al. successfully formulated oleogel with thyme essential oil, designed its optimal formulation, and investigated the influence of ingredients on texture parameters of the preparation while also testing for its antimicrobial activity. The formulation optimization of oleogel was designed by means of a response surface model using Design-Expert 6 program, accompanied by texture analysis. The characterization and quantification of components of essential oils and oleogels were performed by GC-FID. The antimicrobial activity of oleogels with essential thyme oil was determined against* Candida albicans*. Based on the microbiological test, the concentration of 0.05% of thyme essential oil in the oleogel mixture was shown to exhibit an antifungal activity against* Candida albicans.*

Nonalcoholic fatty liver disease (NAFLD) is the most common type of liver disease in developed countries. M. Hong et al. conducted a study to explore the potential protective effects and mechanisms of action of* Gynostemma pentaphyllum *(GP) extract on NAFLD. The* in vivo* results showed that GP extract alleviated fatty degeneration and haptic fibrosis in NAFLD mice. Through a network pharmacology approach and biomedical assay, the potential active components of GP and their intracellular targets for hepatoprotective activity were identified as acyl-CoA oxidase (ACO) and carnitine palmitoyltransferase-1 (CPT-1).

H.-S. Park* et al.* performed a systematic review and meta-analysis of the effects of* Panax ginseng* on obesity in animal models. Sixteen studies were included in the review. The meta-analysis indicated that oral administration of* P. ginseng* is effective against weight gain particularly in the obesity model induced by high-fat diet. The review showed that while* P. ginseng* improved serum lipid profile in animal obesity model, its processing method may affect treatment outcomes.

J. Zhong et al. performed another systematic review to evaluate the efficacy and safety of Ma-Huang-Fu-Zi-Xi-Xin decoction (MHFZXXD) in patients with allergic rhinitis (AR). The review included six randomized control trials (RCTs) in 576 patients. Despite its long use and recommendation in a clinical guideline for the alleviation of symptoms of AR, this systematic review concluded that there is insufficient evidence to support the use of MHFZXXD alone for routine treatment of AR. Several scientific issues were identified in the included RCTs, indicating that there were various types of biases in each study, including selection, performance, attrition, and reporting bias. In addition, the sample size of each trial was shown to be too small to provide conclusive results.

In conclusion, significant progress is seen in the application of Western research methodologies to the evidence-based evaluation of, and further development of, traditional herbal medicines. The research presented in this special issue demonstrates advances in the technologies and scientific strategies for an evidence-based approach to herbal medicines. This offers promise for a more solid and uniformly accepted basis for the confirmation or rejection of the continued use of specific traditional herbal medicines. The employment of these research methodologies also opens new vistas for discovering new chemical compounds and formulations that may support the development of traditional herbal medicine formulations into Western pharmaceuticals. This special issue further provides evidence that modern techniques and methodologies, including mathematical modeling, are not limited in their application to any stage of herbal medicine research and development, assisting both traditional practitioners and Western scientists in addressing challenges to the scientific validity of specific herbal medicines as well as to help further develop these medicines for uses outside their traditional practices and contexts.

